# Mobility-Aware Federated Learning Considering Multiple Networks

**DOI:** 10.3390/s23146286

**Published:** 2023-07-10

**Authors:** Daniel Macedo, Danilo Santos, Angelo Perkusich, Dalton C. G. Valadares

**Affiliations:** 1 Department of Electrical Engineering, Federal University of Campina Grande, Campina Grande 58429-900, Paraiba, Brazil; dalton.valadares@embedded.ufcg.edu.br; 2 Virtus RDI Center, Federal University of Campina Grande, Campina Grande 58429-900, Paraiba, Brazil; danilo.santos@virtus.ufcg.edu.br (D.S.); perkusic@virtus.ufcg.edu.br (A.P.)

**Keywords:** machine learning, distributed learning, federated learning, mobility

## Abstract

Federated learning (*FL*) is a distributed training method for machine learning models (*ML*) that maintain data ownership on users. However, this distributed training approach can lead to variations in efficiency due to user behaviors or characteristics. For instance, mobility can hinder training by causing a client dropout when a device loses connection with other devices on the network. To address this issue, we propose a *FL* coordination algorithm, *MoFeL*, to ensure efficient training even in scenarios with mobility. Furthermore, *MoFeL* evaluates multiple networks with different central servers. To evaluate its effectiveness, we conducted simulation experiments using an image classification application that utilizes machine models trained by a convolutional neural network. The simulation results demonstrate that *MoFeL* outperforms traditional training coordination algorithms in *FL*, with 156.5% more training cycles, in scenarios with high mobility compared to an algorithm that does not consider mobility aspects.

## 1. Introduction

Machine learning (*ML*) is increasingly used in various research areas, including computer vision, decision-making, natural language processing, computer graphics, and intelligent control [1]. In the context of the Internet of Things (IoT), *ML*-based solutions are developed to address network challenges such as traffic engineering, network management, security, internet traffic classification, and computational resource allocation [1]. *ML* is also widely applied in other domains, such as intelligent transportation for optimizing routes and parking [2], human health monitoring [3], and industrial processes [4,5].

Mobile devices have become widespread in the healthcare sector and can store users’ health data [6]. Integrating *ML*-based applications into mobile devices makes it possible to estimate energy expenditure, detect vital signs, and predict sleep quality based on physical activity data collected during waking hours [7,8].

A dataset is essential for effectively training some *ML* applications and algorithms. Additionally, some applications require that data be exclusively accessible to their owners. In healthcare applications, for instance, the sharing of patient data is legally and ethically restricted, posing challenges in data availability [9,10,11].

Another *ML* application scenario that requires data privacy and IoT is autonomous vehicle applications, where *ML* models can be used to perform autonomous driving tasks, and collaboration between vehicles can improve the performance of *ML* algorithms [12,13]. Such collaboration allows autonomous vehicles to exchange sensor data to improve the accuracy of the results for the *ML* [14]. Data security and privacy concerns pose a significant challenge in sharing data between connected vehicles. Without proper protection, there is a risk of malicious interception and unauthorized access to private information [15,16]. Consequently, ensuring data security and privacy becomes crucial for enabling vehicle ML techniques. It is essential to develop strategies that guarantee the privacy of shared data, mitigate potential attacks, and establish a robust framework for the safe implementation of *ML* techniques in vehicles [5,12,17,18].

The *FL* techniques arise to solve the data privacy requirements in machine learning applications. In *FL*, there are two essential roles for learning: the client and the central server. Clients train *ML* models locally, allocating computational resources to do so. In this sense, clients are usually applications’ users [19]. Therefore, in this work, we adopt the term clients to specify application users and the devices that perform local training in *FL*. The central server coordinates the *FL*, selecting the clients to perform local training and aggregating their training results into a single global model. The first *FL* coordination algorithm was named *FedAvg* [20,21]. This paper uses the term network to define the clients connected to a central server, sharing the same global model.

In *FedAvg*, the central server randomly chooses a group of clients and shares the global model with them so that they can perform local training to update and improve the global model. Furthermore, rather than clients sharing the input data for training, as in other distributed *ML* techniques, the clients’ training result is shared with the central server, ensuring clients’ data privacy [22].

A dropout problem can occur during the *FL* training because of the clients’ mobility in the network. This problem can happen if a client is requested to contribute to the model training but does not finish the local training tasks or send its results to the central application. A possible cause for this problem is the clients’ mobility, which can interrupt the communication between the *FL’s* central server and clients, interfering in the training coordination and interrupting the training results sharing.

In a local training cycle, clients can compromise the performance of other tasks when employing computing resources for the training. Thus, the performed processing is useless and lost if there is no communication with the central device. The global model is relatively unaffected from the central server perspective, given that other clients satisfy its requirements by contributing to their local training results. This way, the dropout of a single client only affects the global model once most of the other clients carry out their contributions successfully.

Nevertheless, as the clients’ dropout number increases, the learning of the global model starts to be impacted, decreasing the learning accuracy and convergence. Thus, usual issues of the conventional ML techniques, such as overfitting and underfitting, can also occur in the *FL* scenarios [23]. In the worst case, if the number of clients available in the network for training the model is too small, the learning can become impossible or skewed.

In this paper, based on the traditional *FL* algorithm, *FedAvg* [24], we propose the new *MoFeL* algorithm for multiple central servers simultaneously. *MoFel* differs from *FedAvg* in two steps: the training initialization strategy and the clients’ selection. During the beginning of the model training, *MoFeL* imposes a procedure, initiating a training cycle, while *FedAvg* does not specify what should be adopted. For the clients’ selection, *MoFeL* considers clients with fewer chances to drop out of the training, while *FedAvg* selects clients randomly.

Furthermore, in our new proposal, *MoFeL* evaluates multiple networks with different central servers simultaneously, ensuring that all of them can run the training, which is essential, especially in scenarios with mobility. As far as we know, this is the first *FL* algorithm in the literature that simultaneously addresses the evaluation of multiple networks.

In prior studies, we examined clients’ mobility in *FL* applications as they migrated to different central servers. However, each central server independently solved an optimization problem to select clients, resulting in a solution not coordinated with other central servers. We observed that solving the optimization problem for client selection required significant computational resources, which could pose challenges for central servers with limited computational capabilities [25]. In the current version of *MoFeL*, we consider another profile for the *FL* coordination, named the central station. The central station evaluates a group of central servers simultaneously to guarantee an evenly satisfactory solution for all central servers.

Client selection, including the optimization problem, is the responsibility of the central station. Transferring responsibility for selecting clients from the central server to the central station facilitates allocating computational resources specifically to that single device. This ensures the optimization in choosing clients and reduces resource allocation at the selection of clients in central servers.

The central station receives information from all central servers and clients (e.g., computational resources for local training, clients’ routes, and clients’ time connected to each central server). Based on this information and considering the application requirements, the central station establishes an optimization problem to select the clients for the central servers. This optimization problem can consider the minimum accuracies of global models or other application requirements in this context.

Thus, *MoFeL* is especially important for applications with the following features:

- The clients are mobile;
- The application does not oppose the disclosure of their routes;
- The models need training frequently to update;
- The trained models are different for each central server.

To evaluate the efficiency of *MoFeL*, we carried out experiments through simulations. In this paper, it is essential to note that the term efficiency refers to the capability of the *FL* algorithm to ensure the completion of training cycles while minimizing the computational resources allocated to clients. Additionally, it aims to meet the minimum required model training accuracy specified by the application. The results indicate that *MoFeL* can perform federated training even in scenarios with intense client mobility, while other traditional algorithms for training coordination cannot.

The main contributions of this work are summarized as follows:

- We propose a mobility-aware *FL* algorithm with multiple central servers analysis simultaneously;
- We formalize an optimization model that serves as a benchmark for new proposals;
- We evaluate the proposed technique comparing it with the *FedAvg*.

The remainder of this paper is organized as follows: Section 2 presents related work; Section 3 addresses an example scenario for applying *MoFeL*, highlighting the architecture and motivation for using the *MoFeL*; Section 4 describes the *MoFeL* algorithm and the optimization problem; Section 5 describes the experimental simulations; Section 6 presents and discusses the simulation results; lastly, Section 7 concludes this paper.

## 2. Related Work

Zhang et al. [26] proposed an *FL* algorithm, named *CSFedAvg*, that alleviates the accuracy degradation caused by clients’ non-IID (non-Independent, Identically Distribute) data. Their proposal considers a heterogeneous weight divergence present among the clients’ data. Thus, the algorithm chooses the clients with a lower degree of non-IID data to train the models with higher frequency. The authors conducted simulations, showing that the proposal improves the training performance compared to other *FL* algorithms. Nishio and Yonetani [27] proposed an *FL* algorithm that mitigates clients’ problems with limited computational resources, demanding higher training times, and poor wireless communications requiring longer upload times. Their proposal, named *FedCS*, considers the clients’ resource constraints for selecting the training participants. Additionally, the central server aggregates many updates at once to accelerate performance. The authors performed an experimental evaluation with public image datasets, training deep neural networks in a MEC (Multi-access Edge Computing) environment. The results demonstrate that *FedCS* can reduce the time to complete the training process compared to an original *FL* algorithm.

Although these two works consider mobile applications, they do not consider mobility aspects for client selection procedures.

In this sense, Wang et al. [28] proposed a client selection algorithm with mobility support for vehicular networks, where vehicles have high mobility and frequently switch between regions with different traffic characteristics. The proposal considered an architecture with edge computing, in which vehicles assume the role of clients and edge servers assume the role of a central server coordinating the FL. Besides, the authors also proposed another algorithm for allocating multidimensional communication resources to optimize the cost of *FL* after selecting participants for the training. In this work, the clients’ selection starts with the sharing of vehicle information with the central server, referring to the travelers’ distance within the central server’s domain, vehicle speed in free flow, as well as information about the environment, such as the volume of traffic in the area.

Still, in vehicular scenarios, Li et al. [29] identified that the limited computational resources for training the models locally and the locomotion of the vehicles could lead to low accuracy and high training delays of local models. Thus, the authors proposed a joint optimization scheme in selecting clients to train and allocate resources for the FL. This work uses *FL* in a high-precision FL-based cooperative map caching application to achieve dynamic edge caching while protecting clients’ privacy. In the selection stage, the authors proposed an optimization model considering the communication link, the computational processing capacity, and energy availability. In the solution, if the vehicle has not uploaded information within an established period, the central device does not wait for the training completion of this vehicle, aggregating the local parameters of other vehicles. Even though the work recognizes mobility as an essential factor in its application, it does not consider the mobility characteristics of the vehicles in the clients’ selection. Furthermore, considering the constraint of computational resources, running local training without leveraging its results in the aggregation is frustrating for the client who has committed the computational resources.

Xiao et al. [30] proposed a greedy algorithm to select vehicles for *FL* local training, considering their positions and velocities. The authors described a min-max optimization algorithm that optimizes the computation capability, transmission power, and local model accuracy, achieving the minimum cost for the *FL*. The simulation results demonstrated that the proposal presented good convergence with an acceptable cost. Deveaux et al. [31] considered vehicular mobility to propose an orchestration mechanism for data distribution-aware *FL*. The authors described protocols exchanging training requirements among the entities to improve the model training speed and accuracy. Experiments performed with the *MNIST* dataset presented improvements in the training speed and model accuracy compared to traditional *FL* algorithms.

Considering the impact of client mobility on learning performance, Feng et al. [32] proposed a mobility-aware cluster federated learning for hierarchical federated learning (*HFL*) in wireless networks. In this proposal, the clients move, causing the connection change between edge servers, preventing the conclusion and sharing of the local training results to the central server. The proposed algorithm, called MACFL, enables a new technique for updating the local training and aggregating the global model since the existing aggregation schemes consider the weighted average [20,33], which becomes the bottleneck of performance due to divergences in non-IID data distribution and client mobility.

The studies indicated that the mobility evaluation in *FL* techniques is recent, despite being a decisive factor for their success, including some studies that already consider the client’s mobility. However, these works still do not consider the clients’ routes and destinations. Thus, in the studies presented, the client’s path during migration does not add information for the clients’ selection in the central servers. However, the client route information can be used to improve *FL* if not confidential. The definition of which data must have restricted access to clients is unique to the application.

Thus, this work addresses applications that enable the sharing of client mobility information and is a pioneer in evaluating the selection of clients in *FL* considering several central servers simultaneously. In this way, it is possible to meet the needs of central servers without overloading clients and enabling the selection of clients capable of executing local training, even if the application has mobile clients.

## 3. Background and Motivation

**Figure 1:**
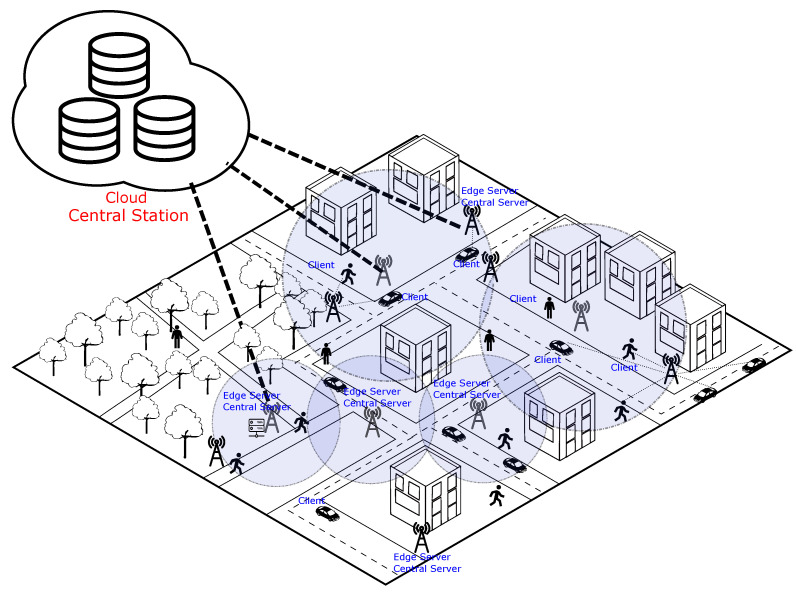
A smart city scenario based on an edge and cloud computing architecture.

To explain the proposed *FL* coordination technique, we present in Figure 1 a smart city scenario based on an edge and cloud computing architecture. The proposed *FL* coordination technique aims to optimize resource usage in a smart city scenario. Figure 1 illustrates an architecture where IoT devices are deployed in vehicles and carried by people in the city. These devices establish wireless connections with various base stations to maintain connectivity with central servers and meet the application’s quality of service requirements.

In the proposed scenario where *FL* is used in edge applications, edge servers allocate central servers, and each central server defines its own global *FL* model. When a device connects to the edge server in the *FL* application, the corresponding client connects to a specific central server. Each central server has its distinct global model independent of the others. Respecting the data confidentiality and ownership principles of *FL*, client data used in local training and the global models within each network cannot be shared among central servers.

A central station in the cloud is connected to all edge servers in the scenario. This central station assists the coordination of *FL*, particularly in the client selection stage. The central station can access information from all clients, such as their displacement routes, speeds, and available computational resources for local model processing. Additionally, the central station is aware of the territorial reach of each central server, precisely defining the geographic area through which clients move when connecting to a particular central server until they switch connections to a new central server.

**Figure 2:**
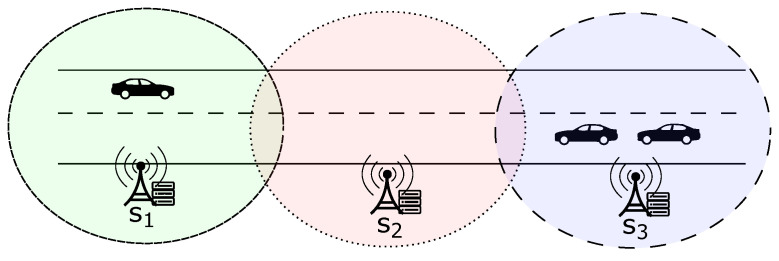
Example of clients traveling between central servers.

To understand the migration of clients among central servers, Figure 2 presents a scenario of vehicle traffic on a highway within a micro-region of the larger smart city depicted in Figure 1, where intelligent vehicles are clients who move between different central servers ($s_{1}$, $s_{2}$, $s_{3}$). In order to model the client migration between central servers, a graph representation can be utilized. In this work, we use the migration term to refer to changing a client’s connection between central servers.

**Figure 3:**
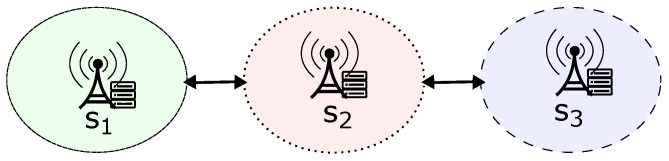
Example of graph referring to Figure 2.

For this purpose, we can consider an undirected graph (V,E) that simulates the traffic of clients between networks. In this graph, *V* represents a set of finite vertices that correspond to the domains of central servers, while *E* represents a finite set of edges defined as (u,v), where u∈V and v∈V. Thus, an edge (v,u) indicates a pathway for a client to migrate from node *v* to node *u* within the graph. It is important to note that each client belongs to a single central server’s domain at any time. As a result, only one graph node can include a client at any particular moment. Thus, the scenario described by Figure 2 can be mapped in the graph of Figure 3.

In specific applications, it is beneficial to maintain different models on central servers to optimize user access based on their connection to a particular server. For instance, vessels exhibit different mobility behaviors in maritime traffic depending on the region they navigate. Each region has fixed obstacles that directly influence navigation, alongside dynamic factors such as moving obstacles (e.g., animals) and changes in environmental conditions like tides, currents, and wind behavior. Machine learning models have been developed to enhance the safety of maritime transport systems by proactively preventing collisions based on region-specific training [34,35,36].

Similar challenges arise in air traffic involving Unmanned Aerial Vehicles (*UAVs*), particularly when flying over cities. Mobility control applications for *UAVs* must account for mobile obstacles, fixed obstacles, and ever-changing environmental conditions that directly impact their flight paths [37].

To address the need for frequent training, the *MoFeL* algorithm continuously evaluates clients, including their mobility patterns, to facilitate training and model updates. If frequent training is not required, the traditional *FedAvg* technique can be used, with the understanding that the model will be trained over the long term, even in challenging scenarios.

Training different models specific to territorial regions and regularly retraining them to adapt to dynamic scenarios is crucial in these contexts. The proposed architecture (refer to Figure 1) caters directly to such applications. Each coverage area can have an edge server deployed, which assigns a central server responsible for training a unique model tailored to the specific characteristics of that coverage area.

Additionally, in scenarios where frequent training is necessary, *MoFeL* is a practical algorithm that continuously evaluates clients, including their mobility patterns, to enable regular training and model updates. However, for applications where frequent training is not required, the traditional *FedAvg* technique can be used, ensuring that the model is trained in the long term, even in challenging conditions. For example, in urban environments where traffic conditions change over time, continuously updating models ensures the efficiency of public transportation systems [38].

## 4. *MoFeL*

This section provides an overview of the *MoFeL* algorithm, emphasizing the roles of clients, central servers, and the central station. The symbols frequently used in this paper are summarized in Table 1.

In the *MoFeL* design, we consider a set *N* of clients that move within a network and connect with central servers grouped in the set *S*. Each client *n* (n∈N) has a specific time requirement $r_{n}$ for local training and moves at a certain speed $v_{n}$ ($v_{n}\in R$). It is important to note that the client’s speed ($v_{n}$) and the time required for training $r_{n}$ ($r_{n}\in R$) are inherent characteristics of the client that remain constant regardless of its connection to a central server or geolocation.

On the other hand, each central server *s* (s∈S) covers a specific territorial area $a_{s}$ ($a_{s}\in R$). A central server’s territorial reach ($a_{s}$) is a characteristic unique to that server, constant, and independent of client connections. Whenever a client *n* connects to a central server, it must traverse the corresponding territorial area until changing its connection. Therefore, a client *n* loses connection with a central server *s* after staying connected $a_{s}/v_{n}$ time units.

Consequently, a client *n* can only perform local training within the network managed by a specific central server *s* if and only if $a_{s}/v_{n}\geq r_{n}$. The problem formulation and mathematical modeling can be further expanded by incorporating more complexity in variables $a_{s}$, $r_{n}$, and $v_{n}$. For simplicity in understanding the optimization problem’s modeling, we assume constant values for these variables in this work.

**Figure 4:**
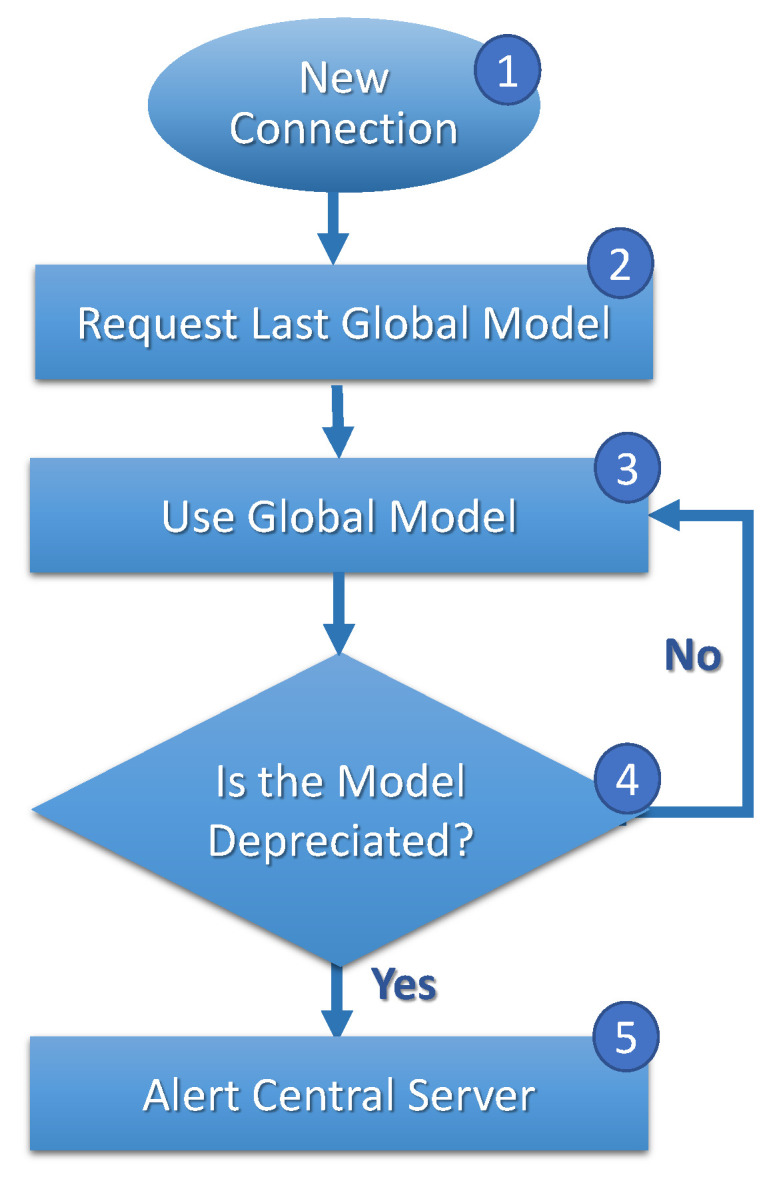
Clients’ *FL* cycle.

**Figure 5:**
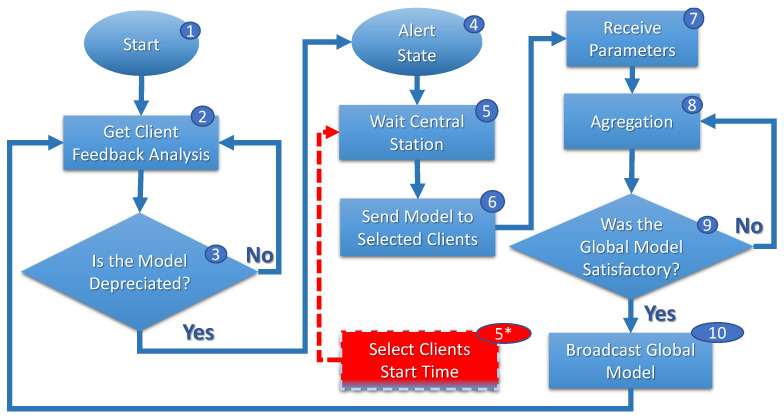
Central servers’ *FL* cycle.

**Figure 6:**
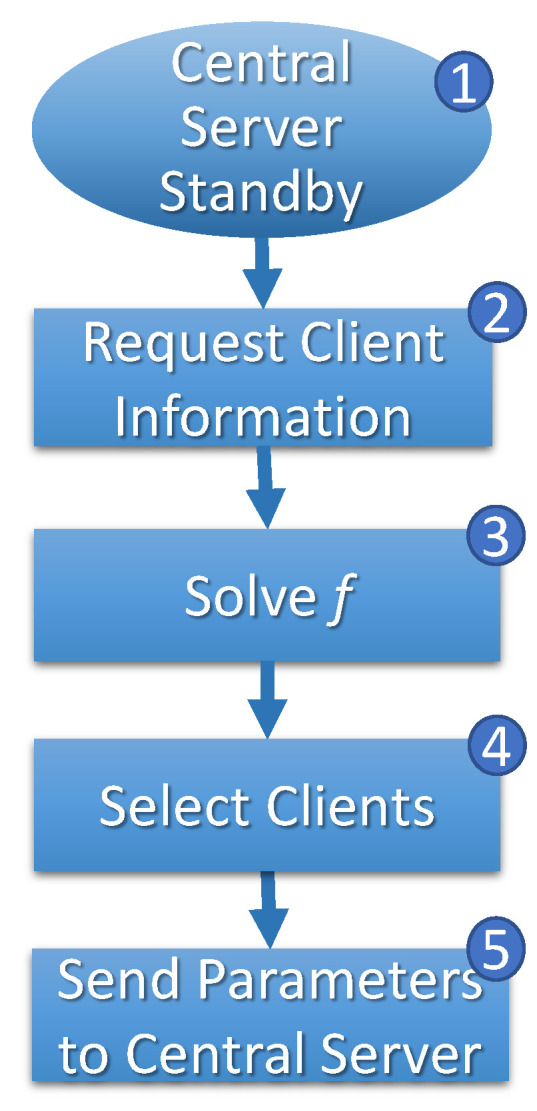
Central station’ *FL* cycle.

Figure 4, Figure 5 and Figure 6 show *FL* execution cycles on clients, central servers, and the central station. In Figure 4, when migrating and connecting a new central server (step 1), the client requests a current global model (step 2) from the central server. The client evaluates the model frequently (step 3), and if the client evaluates that the model is deprecated (step 4), the client reports to the central server (step 5).

The model’s inefficacy can be evaluated from sensitivity, specificity, accuracy, precision, and others [39]. For example, it is possible for applications to consider the average and the standard deviation of the accuracies in the model training evaluation, analyzing a maximum threshold for the standard deviation and a minimum threshold for the average value. In this way, a central server requests the accuracy values from the clients to calculate the mean and standard deviation and decides whether to update the model training if it does not meet the established condition. The definition of the minimum threshold for the average accuracy and the maximum threshold for the standard deviation depends on application requirements.

In any case, the central server enters an alert state (step 4, Figure 5) to start training the *FL*, waiting for information about selected clients by central stations (step 5, Figure 5). When a central server requires model retraining in the alert state, it notifies the central station accordingly. As illustrated in Figure 6, the central station awaits requests from other central servers within a predefined interval (step 1). Once all requests are received, the central station gathers information from all clients (step 2), including their respective routes and speeds ($v_{n}$). Utilizing this information, the optimization problem, represented by Equation (1), is initiated (steps 3 and 4) to determine the optimal solution.

The evaluation process is conducted in a predictive manner, considering a future time interval denoted as $\phi =[t_{i},t_{f}]$. This interval begins when the central servers enter an alert state ($t_{i}$) and extends until time $t_{f}$. The solution to Equation (1) determines when and which clients participate in the local training process for each central server that has requested to update its global model.

Once the selection is determined, the central station promptly notifies the respective central servers (step 5, Figure 6). In turn, the central server awaits the definition of the selection of clients by the central station (steps 5 and 5*, Figure 5). With the report, the central server waits when the selected clients connect to its network to start training.

Each central server’s training cycle is scheduled to begin once all designated clients have connected to its domain, as depicted in steps 7, 8, 9, and 10 of Figure 5. The central station assesses an objective function (Equation (1)) to identify a result that is deemed minimally satisfactory. This analysis helps to determine the optimal moment to initiate the training process.

The selection of clients to participate in the local training is based on the following criteria:

(1) $$\begin{array}{c} f=\min(\sum_{s\in S}F_{s}) \end{array}$$

In Equation (1), $F_{s}$ is the global model error function aggregated by the central server *s*. Thus, the objective of Equation (1) is minimizing the sum of all error functions for the models aggregated by each central server. In detriment of the panoramic evaluation of all the networks’ models, restrictions can be attributed in this equation solution to direct the focus of fairness to it, depending on the application, such as controlling the number of training executed individually by the clients, controlling the number of clients participating in training, and minimizing the training time or another application constraint.

The value of $F_{s}$ is defined with a complete training of the model. Therefore, the solution to the proposed optimization problem must predict $F_{s}$ before the final decision on the selection of clients. The prediction of $F_{s}$ is analyzed based on the characteristics of the application, the database, and the training technique used in the application. In this work, based on the simulation and application in the following sections, we adopted the inference of $F_{s}$ through the number of clients that will contribute to the local training. In future works, we will analyze how the prediction of $F_{s}$ interferes with the solution of the optimization problem.

Equation (1) has mandatory constraints, by the Equations (2), (4), (5), (7) and (8), that are detailed as follows. Equation (2) is a logical constraint and defines that if a client *n* is selected for the central server *s* for local training, at least one time in interval ϕ, *n* participates in the training cycle of *s*. Equation (2) is given as follows:

(2) $$\begin{array}{c} \forall s,\forall n,\forall t,\;x_{sn}-z_{snt}\geq 0 \end{array}$$

where $x_{sn}$ is a binary variable, such as $x_{sn}\in \left\{0,1\right\}$, with $x_{sn}=1$ denoting that the client *n* was chosen for local training and $x_{sn}=0$ otherwise. *t* is the time step, such that t∈ϕ. $z_{snt}$ is a binary variable, such as $z_{snt}\in \left\{0,1\right\}$, and indicates whether the client *n* is participating in the local training at the instant *t*. As with $x_{sn}$, the variable $z_{nt}$ is the result of the Equation (1) solution and takes on the values:

(3) $$z_{snt}\in \{0,1\},z_{snt}=\{\begin{array}{cc} 1, & \mathrm{if}\mathrm{client}n\mathrm{is}\mathrm{training}\mathrm{in}\mathrm{central}\mathrm{server}s\mathrm{at}\mathrm{time}t.\\ 0, & \mathrm{if}\mathrm{client}n\mathrm{is}\mathrm{not}\mathrm{training}\mathrm{in}\mathrm{central}\mathrm{server}s\mathrm{at}\mathrm{time}t. \end{array}$$

The constraint in Equation (4) collaborates with the definition that all selected clients must remain connected and available computational resources during the *FL* cycle. The Equation (4) must be applied when condition $\sum_{n\in N}z_{snt}>0$ is true. The Equation (4) and the Equation (2) define together that $x_{sn}=1\Leftrightarrow \sum_{t=t_{i}}^{t_{f}}z_{snt}\geq 1$, thus

(4) $$\forall s,\forall t,\sum_{n\in N}x_{sn}=\sum_{n\in N}z_{snt}$$

The constraint in Equation (5) guarantees that a client *n* can only participate in a training cycle in central server *s* while *n* is connected to *s*, i.e., $z_{snt}=1\Rightarrow y_{snt}=1$.

(5) $$\begin{array}{c} \forall s,\forall n,\forall t,\;(y_{snt}-z_{snt})\geq 0 \end{array}$$

where $y_{snt}$ is a binary variable, such as $y_{snt}\in \left\{0,1\right\}$. $y_{snt}$ is inherent to the client’s mobility and defines whether client *n* is connected to the central server *s*. Therefore, $y_{snt}$ is defined by the route and speed of each client, in addition to $a_{s}$ of the central servers belonging to the client’s route. So, $y_{snt}$ is defined as:

(6) $$y_{snt}\in \{0,1\},y_{snt}=\{\begin{array}{cc} 1, & \mathrm{if}\mathrm{client}n\mathrm{is}\mathrm{connected}\mathrm{to}\mathrm{the}\mathrm{central}\mathrm{server}s\mathrm{at}\mathrm{time}t.\\ 0, & \mathrm{if}\mathrm{client}n\mathrm{is}\mathrm{not}\mathrm{connected}\mathrm{to}\mathrm{the}\mathrm{central}\mathrm{server}s\mathrm{at}\mathrm{time}t. \end{array}$$

The constraint in Equation (7) defines the execution of local training to be continuous, i.e., without interruptions. Therefore, Equation (7) is directly related to the Equation (4).

(7) $$\begin{array}{c} \forall s,\forall n,\;\min_{t=t_{i}}^{t_{f}}\left(z_{snt}\cdot t\right)+x_{sn}\cdot \max_{n\in N}\left(x_{sn}\cdot r_{n}\right)=\max_{t=t_{i}}^{t_{f}}\left(z_{snt}\cdot t\right) \end{array}$$

Equation (8) defines a constraint that guarantees that a client *n*, selected by *s*, must be available during the time required to complete all training cycles. *MoFeL* requires the aggregation of results of all local training. Therefore, the time required for completion is defined by the slowest client running the local training ($\max_{n\in N}\left(x_{sn}\cdot r_{n}\right)$).

(8) $$\begin{array}{c} \forall s,\forall n,\;x_{sn}\cdot \sum_{t=t_{i}}^{t_{f}}z_{snt}\geq \max_{n\in N}\left(x_{sn}\cdot r_{n}\right) \end{array}$$

After determining the starting point of training, the clients with $x_{sn}=1$ are selected, while the remaining steps (7, 8, 9, and 10) of the algorithm are depicted in Figure 5 follow a similar approach to the *FedAvg* algorithm.

Besides the previously defined restrictions, it is possible to expand the modeling of the solution presented in Equation (1) by incorporating additional constraints to promote fairness requirements based on the specific application. For instance, Equation (9) introduces the constraint that the standard deviation of the error functions between the models defined in the central servers must be smaller than a threshold value (γ), ensuring that the data remains close to a predetermined average. By including such restrictions, the optimization process can be tailored to meet fairness objectives in distributing the global model’s accuracy among the central servers.

(9) $$\begin{array}{c} \sqrt{\frac{\sum_{s\in S}{\left(F_{s}-\bar{F_{s}}\right)}^{2}}{∥S∥}}<\gamma \end{array}$$

where γ is a constant defined in the optimization problem based on application requirements and $\bar{F_{s}}$ is the average of all $F_{s}$.

Another example of a restriction is presented in Equation (10). This restriction limits the number of training sessions (*n*) that each client can perform, ensuring that it does not exceed a certain threshold (δ). This constraint is implemented to prevent training overload and excessive allocation of computational resources to specific clients. By setting this restriction, a more balanced allocation of training tasks can be achieved to avoid client overload when selecting the same clients multiple times to run local training on different central servers.

(10) $$\begin{array}{c} \forall n,\;\sum_{s\in S}x_{sn}\leq \delta \end{array}$$

It is crucial to emphasize that as restrictions are added to modeling an optimization problem, the feasibility of finding a solution may be compromised due to the reduction in the set of potential solutions. Balancing the incorporation of necessary constraints while maintaining a feasible solution space becomes a significant challenge in modeling the problem to meet the application’s specific requirements. Striking the right balance is essential to ensure the optimization problem remains solvable and effectively addresses the application’s constraints.

## 5. Experimental Evaluation

For the experimental evaluation, we constructed a simulated *FL* edge computing environment comprising central servers and clients and defined a network with a set of clients and a central server. In the *FL* process, we implemented the on-device training using the TensorFlow Federated framework [40], a widely used machine learning library. The simulation utilized the *MNIST* dataset, commonly used for handwritten digit recognition tasks, with images and corresponding labels for training and testing machine learning models [41].

In the simulation, each client is assigned a unique subset of the handwriting database from *MNIST*. Initially, the client does not have immediate access to its dataset slice, and the samples are uniformly distributed during the simulation. As the simulation progresses, the client’s database gradually increases in size.

Additionally, clients have the ability to migrate randomly within the network, based on a uniform distribution. The simulated application focuses on classifying images.

In the following subsections, we discuss the methodology used in the execution of the simulation experiment (Section 5.1) and the simulation parameters (Section 5.2).

### 5.1. Experimental Methodology

**Figure 7:**
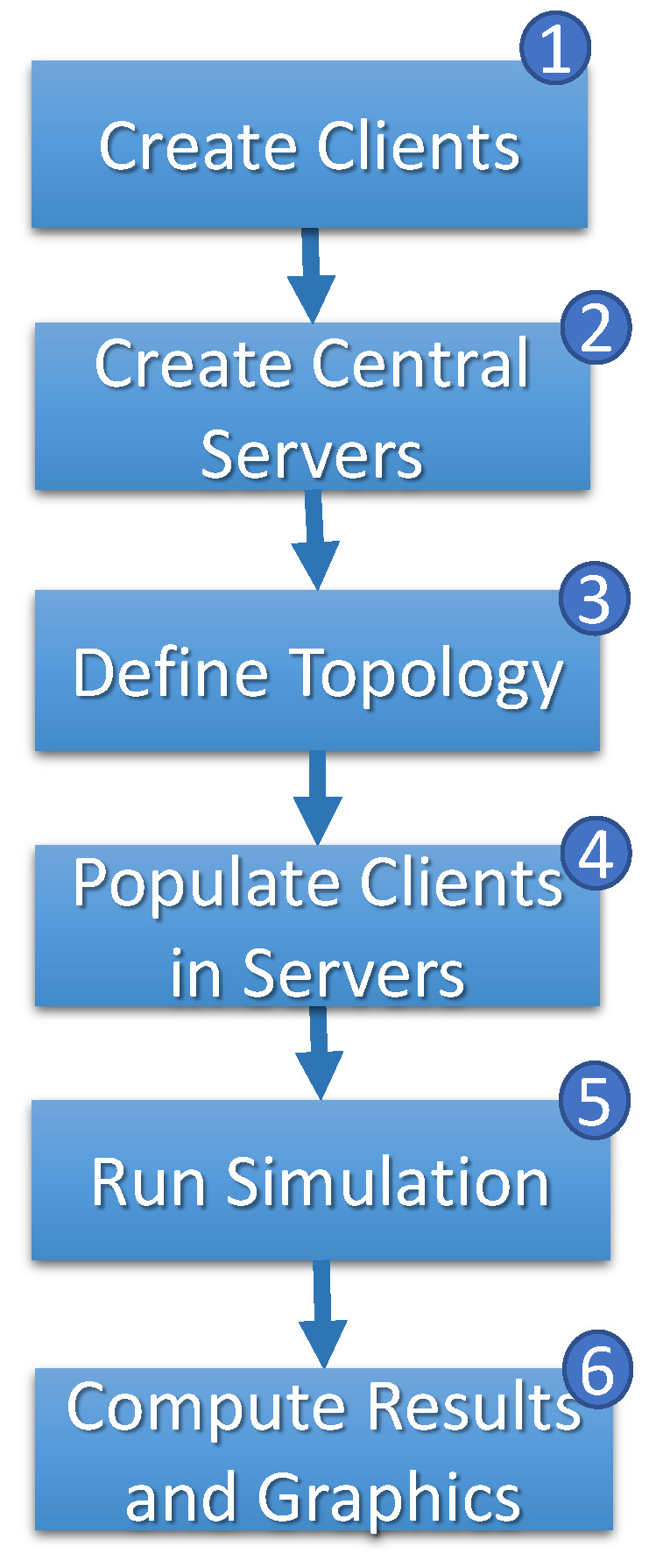
Simulation flow.

Figure 7 shows the simulation flowchart. The first step (step 1) generates ∥N∥ clients. For each client, the speed ($v_{n}$) and the time required ($r_{n}$) are randomly chosen, respectively, between $\left[v_{min},v_{max}\right]$ and $\left[r_{min},r_{max}\right]$. The combination between $v_{n}$ and $r_{n}$ of each client represents the system’s heterogeneity.

In step 2, ∥S∥ central servers are generated, and each central server randomly receives the length of the path to be traversed in the network between $\left[a_{min},a_{max}\right]$. With the clients and central servers created, the simulation receives the graph’s structure topology (step 3) as a parameter and randomly connects the clients to the central servers (step 4). When the simulation starts, a timer is initiated on step 5 to track the discrete progression of time *t* (t∈N) throughout the simulation period [0,T]. The duration of the simulation, *T*, is a required parameter to provide.

### 5.2. Experimental Evaluation

The evaluation of *MoFeL* is based on comparing two other proposals for simpler algorithms. The two algorithms are described as follows.

Algorithm $e_{1}$ randomly defines clients’ participation in a training cycle. The selection step does not consider features of the device’s mobility aspects or computational capacity. Thus, it is similar to *FedAvg*. A cycle starts considering fixed time intervals.

Algorithm $e_{2}$ selects clients that can complete the training before they migrate. Therefore, $e_{2}$ considers mobility aspects. However, each central server only evaluates connected clients at the beginning of the training cycle. In turn, the *MoFeL* algorithm takes a different approach. It assesses all clients through the central station to determine the most suitable set of clients for each central server. In *MoFeL*, the central station can select a client for a central server even if the client is not currently connected to that server. The only requirement is that the client establishes a connection with the server at the beginning of the training. This ability to anticipate client selection is made possible by *MoFeL*’s knowledge of the client’s route, allowing it to predict future migrations.

Finally, we evaluated two metrics. First, the number of training cycles (*NTC*) performed. Second, the number of frustrated clients (*NFC*), i.e., the total number of clients who initiated training but failed to complete it. Additionally, we evaluated the training accuracy average and the standard deviation of accuracy graphically.

### 5.3. Experimental Setup

For this simulation, we considered a scenario with 4 central servers connected through a mesh topology and 100 clients. In the simulation, $v_{min}$ = 1 m/min, the minimum accuracy that each central server wants to achieve is 0.95, and the *FL* process is requested by the central server until the global model reaches the minimum accuracy in training.

This experiment changes the value of $v_{max}$, and the other simulation parameters are constant. The other simulation parameters are $a_{min}$ = 50 m, $a_{max}$ = 100 m, $r_{min}$ = 1 min, $r_{max}$ = 20 min, and *T* = 2000 min. The simulator generates new clients by changing the value of $v_{max}$ because each client can assume other speeds ($v_{n}$). Increasing the value of $v_{max}$ increases the number of clients with greater mobility migrating between networks more frequently.

We simulated an image classification application using a Convolutional Neural Network, specifically the standard *LeNet-5* [42]. Arbitrarily, we adopted the execution of 10 epochs in local training. All the simulations are performed on a computer with 32 GB RAM memory and a Intel i7-7700 3.60 GHz processor.

## 6. Experimental Results and Discussion

**Figure 8:**
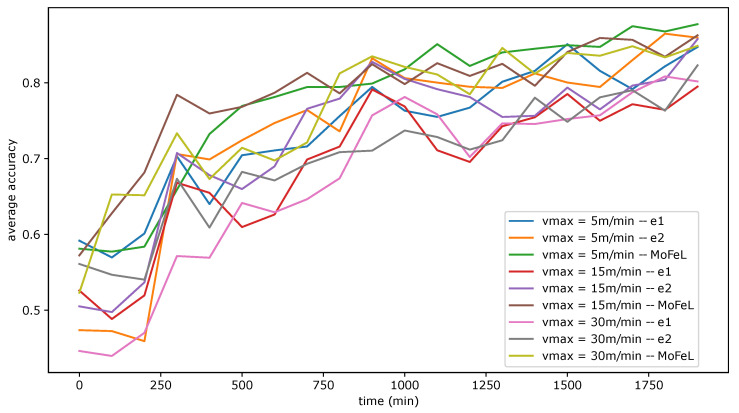
Average between the training accuracies of the central servers.

In this experiment, we evaluated three scenarios: $v_{max}$ = 5 m/min, $v_{max}$ = 15 m/min, and $v_{max}$ = 30 m/min. Figure 8 shows the progress of the average training accuracy across central servers by the number of *FL* simulation steps for each algorithm. Each curve represents the average training accuracy for the combination of an algorithm and a scenario.

Analyzing Figure 8, we can verify that all the algorithms suffered impacts with the increase in the system mobility level, resulting in the worsening of the global models training. This analysis alone supports the importance of evaluating mobility in *FL* since this fact can make model training unfeasible, especially when the mobility level is high. Furthermore, algorithms $e_{2}$ and *MoFeL* suffered losses in accuracy during training as $v_{max}$ increases. However, *MoFeL* was more robust to change scenarios, achieving training with more satisfactory results. In this sense, the experiment corroborates other studies by concluding that a biased client selection brings benefits to *FL* or that the client selection process can consider mobility aspects [26,27,28,29,30,31]. There are two reasons why this happens:

- Increased likelihood of choosing unsuitable clients for training, i.e., unable to complete training before changing their central server connection;
- The lack of clients capable of performing the full training.

Regarding the first reason, as $v_{max}$ increases, the speed average of devices also increases. Thus, algorithms that arbitrarily select clients are more likely to choose clients with greater mobility. In this context, as the level of mobility increases, the number of clients who follow the expression $(r_{n}-a_{s}/v_{n})>0$ increases. The absence of clients capable of training on any central server, i.e., $\forall s,\sum_{n\in N}\left(r_{n}-a_{s}/v_{n}>0\right)=∥N∥$, makes *FL* completely unfeasible.

For algorithm $e_{1}$, it is possible to identify a significant worsening in the model’s accuracy as the speed $v_{max}$ increases. Considering the graphical analysis, clients’ mobility can make the *FL* unfeasible if the training strategies do not consider the mobility aspect during the client selection.

Table 2 summarizes the experiment’s NTC and NFC results for each combination between mobility scenarios and algorithms. Through the NFC values and the graph in Figure 8, it is possible to conclude that the algorithm $e_{1}$ wrongly chose many clients in the scenario with less mobility, causing damage to the application and the clients who made resources available for this training.

Algorithm $e_{2}$ achieved satisfactory results with the variable $v_{max}$ = 5 m/min. However, increasing $v_{max}$ decreases the number of complete training cycles. Even so, the technique ensures that clients unable to complete the training are not selected and, therefore, NFC=0 in all scenarios, like *MoFeL*. Although no client has unnecessarily allocated resources, the application is compromised by inadequate training and model accuracy due to the inability of the central servers to analyze a longer time interval to evaluate clients that will connect to the central servers in the future, i.e., the algorithm $e_{2}$ limits itself to querying the clients connected to it at the query instant. *MoFeL* and $e_{2}$ are biased in client selection strategies. However, in *MoFeL*, a central station analyzes all the clients and all central servers.

Furthermore, the limited view of the central servers in the algorithms $e_{1}$ and $e_{2}$ makes it challenging to adopt equality criteria among clients. In this way, clients with less mobility may be overwhelmed by running more training cycles. *MoFeL* solves this problem by adopting more restrictions in Equation (1). It is possible to propose that, in algorithms $e_{1}$ and $e_{2}$, clients notify the central server of their history of local training so that the central server considers this in selecting clients. However, this proposal would still be ineffective compared to *MoFeL* since the view of the algorithms $e_{1}$ and $e_{2}$ are limited only to clients connected to the central server.

Results in Table 2 show that all algorithms were successful in executing training cycles in at least one of the central servers (NTC>0). However, the number of trained cycles was not the same. Also, when the experiment increased the mobility, *MoFeL* was more robust. Between extreme scenarios, $v_{max}$ = 5 m/min and $v_{max}$ = 30 m/min, the NTC decreased significantly. In algorithm $e_{1}$, the NTC decreased 35.21%, and in algorithm $e_{2}$, it decreased 11%. In addition, there was an increase of 2.4% of NFC in the $e_{1}$ algorithm, that is, an increase in dissatisfied clients for having committed their resources unnecessarily. In turn, the NTC of *MoFeL* remained practically constant.

In Table 2, it is possible to observe an increase in *NTC* in *MoFeL* between $v_{max}$ = 5 m/min and $v_{max}$ = 30 m/min. Despite the increase in mobility between scenarios, *MoFeL* performed more training cycles to achieve a more accurate model. However, with the decrease in available clients to complete the training, the model’s training did not obtain a satisfactory result, as shown in Figure 8. The increase in the *NTC* also occurred in algorithm $e_{1}$. However, this increase was not able to improve the result of training and provoked an increase in *NFC*.

Regarding the number of training cycles, in the scenario with $v_{max}$ = 30 m/min, the *MoFeL* algorithm performed 156.5% training cycles more than $e_{1}$ and 21.64% more than $e_{2}$, demonstrating the efficiency of *MoFeL* in defining instants and clients to execute the training in scenarios with high mobility. In scenarios with less mobility ($v_{max}$ = 5 m/min), there was an increase of 57.7% in the execution of training cycles compared to $e_{1}$ and 2.75% compared to $e_{2}$. Thus, *MoFeL* could perform more training in scenarios with less mobility.

**Figure 9:**
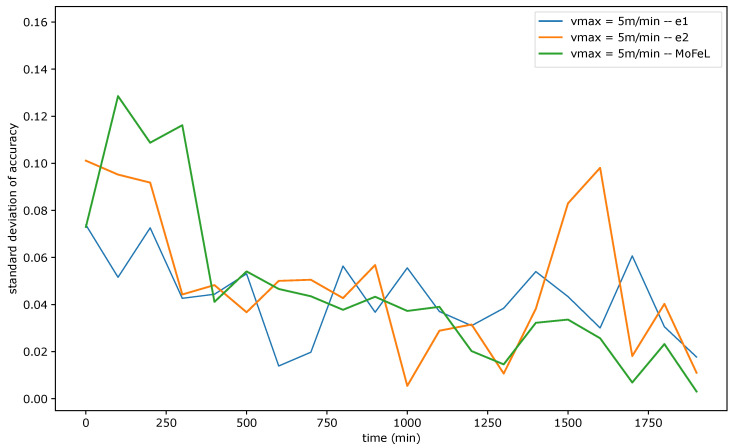
Standard deviation of accuracy for $v_{max}$ = 5 m/min.

**Figure 10:**
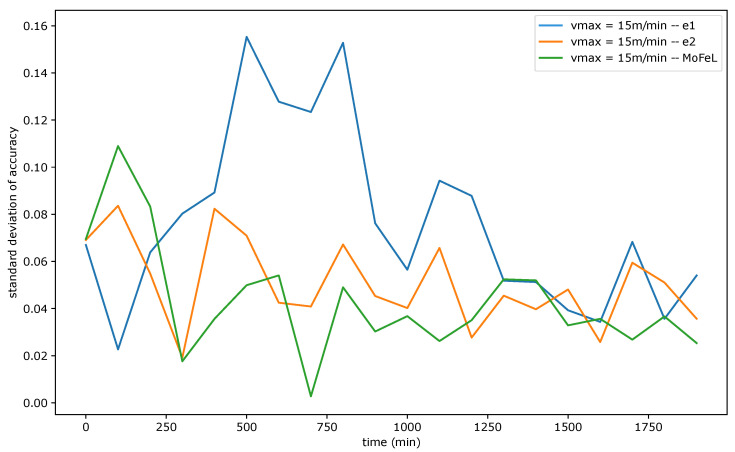
Standard deviation of accuracy for $v_{max}$ = 15 m/min.

**Figure 11:**
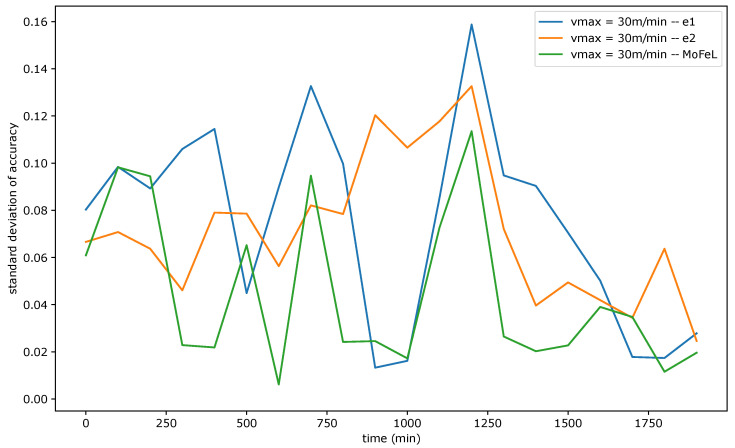
Standard deviation of accuracy for $v_{max}$ = 30 m/min.

Figure 9, Figure 10 and Figure 11 show the standard deviation of training accuracy over time for each algorithm and each mobility scenario. Specifically, Figure 9 represents the scenario with $v_{max}$ = 5 m/min, Figure 10 represents the scenario with $v_{max}$ = 15 m/min, and Figure 11 represents the scenario $v_{max}$ = 30 m/min.

The standard deviation analysis assesses the degree of variation in training accuracy results among different models. When the standard deviation is slight, and the average accuracy is close to the application requirement, the accuracy values are tightly clustered around the average, and most central servers successfully train the global model. However, if the standard deviation is low and the mean accuracy is low, it implies that the training of most servers was not efficient.

Upon analyzing Figure 9, Figure 10 and Figure 11, it is evident that the standard deviation of *MoFeL* is lower than that of the other algorithms for the majority of the simulation duration. Additionally, in Figure 8, it can be observed that the average accuracy of *MoFeL* is higher compared to the other algorithms, indicating that the training results of *MoFeL* are more reliable and effective for most of the servers involved in the simulation. The standard deviation for algorithms $e_{1}$ and $e_{2}$ remained higher than that of *MoFeL* in most mobility scenarios, as indicated by the graphs, indicating that these algorithms favor specific central servers while hindering the training of others. Consequently, algorithms $e_{1}$ and $e_{2}$ proved inefficient for multiple edges and mobility scenarios.

In all scenarios, it is evident that during the initial stages of the simulation (from 0 min to 250 min), the *MoFeL* algorithm exhibits a more substantial variation in standard deviation compared to the rest of the simulation. The variation observed can be attributed to the fact that the global model of each central server is freshly trained and has undergone only a few training cycles and to the fact the central servers wait for the determined instant by the central station to execute the first training. Consequently, the accuracy of the models trained on each central server may experience more oscillations initially. However, this initial variation is eventually overcome as time advances.

Finally, all algorithms consistently exhibited similar standard deviation results across various mobility scenarios, indicating convergence. This convergence occurs because, over an extended simulation period, mobile clients capable of completing local training on each central server have already contributed to the global model definition at some point. Moreover, in the specified simulation methodology, the client database is continually expanded. Consequently, when clients train the data model locally, the older training data continues to be utilized, although its significance diminishes as the simulation progresses. This phenomenon contributes to the convergence of models, as the growing client database in the simulated scenario promotes the stabilization of local training.

The efficiency of *MoFeL* depends on the time interval θ, where $\theta =t_{f}-t_{i}$, for evaluating the solution to Equation (1). A larger θ allows for a broader search range to find a solution, but it also delays the start of training by the central servers. Consequently, clients dissatisfied with the central server model may have already migrated, leading to inconsistencies in model retraining. Increasing θ also increases computational costs for solving Equation (1). Conversely, decreasing θ reduces computational costs but provides a smaller time window for a more suitable solution. Therefore, future work will focus on analyzing and proposing solutions to address these issues.

The solution of Equation (1) presents a challenge to implementing *MoFeL* due to its computational complexity. As the number of clients (|N|) or the number of central servers (|S|) increases, the feasibility of solving Equation (1) becomes impractical. Initially, this work assumes that the central station installed in the cloud can handle the client selection process, making *MoFeL* viable. However, alternative strategies can be employed to overcome this challenge besides relying on sufficient computing resources in the cloud:

- Adoption of optimization techniques that find viable and approximate solutions instead of seeking only the exact solution. For this, the use of techniques, such as genetic algorithms, can approximate the resolution of Equation (1);
- Analysis of mobility behavior patterns can decrease the number of calculations in route inference, as they store repetitive behaviors of clients [43].

Adopting the listed strategies can directly affect the efficiency of the *FL* coordination algorithm since they will bring approximate solutions to Equation (1). In this sense, the application requirements will define whether the error of the solution found is feasible or not, considering the computational gain in solving the problem.

Another disadvantage of *MoFeL* is the dependence on client information, such as computing resource capacity, mobility characteristics, and individual routes. Applications or clients may restrict access and disclosure of this information to the central station as privacy constraints.

Again, it is possible to evaluate strategies to bypass these barriers in the implementation of *MoFel*, giving up an optimal solution to find a viable solution for the requirements and constraints of the application. Some proposed strategies are

- Mobility data evaluation only from a subset of clients who are willing to collaborate with the algorithm or who are interested in application rewards [44,45];
- Approximate and infer the clients’ routes through the observatory perspective of the central server, exempting the client from providing its route with precision.

Despite the implementation’s challenges, the *MoFel* can serve as a benchmark for future improvements and tailoring the algorithm to meet specific application requirements.

## 7. Conclusions

In this work, we presented an *FL* algorithm named *MoFeL* that uses clients’ mobility data at the client selection stage to mitigate damages in the model learning process when dropouts occur during training. In *MoFeL*, the client selection stage is based on the computing resources available and mobility features. Unlike the other algorithms, *MoFeL* simultaneously evaluates different central servers, enabling all central servers to run *FL* in scenarios with mobility.

*MoFeL*’s simultaneous view of multiple central servers allows the application to impose requirements to ensure adequate training based on its criteria. For example, the mathematical model of *MoFeL* can be expanded to minimize the difference between the amount of training performed by each client. Furthermore, the approach of *MoFeL* presents a mathematical optimization model, which can be helpful as a benchmark for other solutions.

The experimental evaluation in this study showed that, in scenarios with high mobility, *MoFeL* had training results with better accuracy when compared to the other techniques ($e_{1}$ and $e_{2}$). Furthermore, an advantage of *MoFeL* is the guarantee not to select unable clients. Thus, clients do not spend computational resources unnecessarily on training since they will not participate in the global model.

The implementation of the *MoFeL* algorithm faces challenges, particularly in dealing with the computational complexity of solving Equation 1. Thus, future research will explore the computational complexity of the *MoFeL* to ensure a more robust implementation. The computational analysis of *MoFeL* will be evaluated, and the simulation methodology will be expanded to examine the relationship between the overhead of client selection and the algorithm’s ability to meet the application requirements. Additionally, the methodology will be extended to include other databases and various *ML* methods to assess the impact of different models on *FL* in mobile scenarios.

Another challenge arises when applications require information about clients’ routes who consider these data confidential or refuse to provide it in advance. Some proposals suggest inferring clients’ mobility information solely from the perception of central servers observing their connected clients. Alternatively, offering incentives to clients who willingly share their information can also be considered. Future research will focus on studying the feasibility of these proposals and analyzing their impact on *FL*.

## Figures and Tables

**Table 1:** Symbols and description.

*N*	Set of clients
∥N∥	Number of clients
*n*	Any client, such as n∈N
$v_{n}$	Client speed, such as $v_{n}\in R$
$r_{n}$	Time required for a client to perform onsite training, such as $r_{n}\in R$
*S*	Set of central server
∥S∥	Number of central servers
$a_{s}$	Length of path to be traversed by any client within a central server

**Table 2:** *NFC* and *NTC* of experiment. The dimension of the variable $v_{max}$ is m/min.

Algorithm	*NTC*			*NFC*		
** $e_{1}$ **	71	39	46	873	881	894
** $e_{2}$ **	109	103	97	0	0	0
***MoFeL***	112	109	118	0	0	0
	** $v_{max}=5$ **	** $v_{max}=15$ **	** $v_{max}=30$ **	** $v_{max}=5$ **	** $v_{max}=15$ **	** $v_{max}=30$ **
